# Supplementary material for: Medial pivot designs result in improved patient reported outcome measures and range of motion when compared to cruciate retaining total knee replacements: A systematic review and meta-analysis
Source: PLoS One. 2026 Feb 9;21(2):e0332548. doi: 10.1371/journal.pone.0332548 (PMC12885310; doi:10.1371/journal.pone.0332548)
Supplement: Attachment — Submitted filename: Response to Reviewers final.docx [file pone.0332548.s002.docx]

**Response to Reviewers**

Dear reviewers,

We sincerely thank you for your time and input reviewing our manuscript. We have considered all points raised and incorporated your valuable input into the revised version. Please see below our responses to each point raised.

**Reviewer 1:**

1. **Reviewer:** Could be made clearer in the Introduction re: when the most recent systematic reviews were published, to help justify this new review

**Response**: We thank you for this suggestion and have clarified the publications years of the quoted evidence accordingly.

2. **Reviewer**: AMPQQ acronym (and all acronyms, for that matter) needs to be defined at first use

**Response**: Noted, thank you for highlighting this, AMQPP now defined within text.

3. **Reviewer**: Inclusion criteria included studies of high quality. Therefore, studies underwent critical appraisal then low quality studies were excluded? I do not recommend this approach. Essentially this constitutes a sensitivity analysis from the outset

**Response**: Whilst we appreciate the concerns regarding the inclusion criteria, this approach was agreed with all authors within the group in order to draw conclusions only from robust, unbiased trials. Such an approach is common within this field and as such we feel this is acceptable. Although this does potentially constitute a sensitivity analysis, no study ended up being excluded on the basis of quality alone.

4. **Reviewer**: Related to this: critical appraisal should be in Results, not Methods. I understand why it is currently in the Methods section, but I disagree this methodological choice (i.e. using critical appraisal as part of the article screening process). A systematic review should include all relevant literature, and then the low quality studies can potentially be excluded in a sensitivity analysis but would still be included in the review overall.

**Response**: Many thanks for highlighting this concern. Text and tables relative to critical appraisal of identified studies has been moved to the results to avoid confusion relating to our methodology.

5. **Reviewer**: Was forward and backward citation chasing conducted?

**Response**: Yes, forward and backwards citation chasing was conducted.

6. **Reviewer**: Has the search been re-run since July 2024? It has now been 12 months so this would be recommended.

**Response**: The search was repeated on 22/10/2025 and summarised in the PRISMA diagram. 25 new potentially eligible articles were retrieved, with 24 excluded on abstract screening. One article underwent full text screening however was excluded on the basis of data reporting methodology.

7. **Reviewer**: I recommend reporting prediction intervals and emphasising these over i-squared values when reporting on heterogeneity. Relevant references: Borenstein 2023 - Avoiding common mistakes in meta-analysis: Understanding the distinct roles of Q, I-squared,tau-squared, and the prediction interval in reporting heterogeneity; IntHout 2016 - Plea for routinely presenting prediction intervals in meta-analysis

**Response**: Thank you for highlighting this to us, we have since calculated the prediction intervals and included these within the results section. Due to the fact that I^2^ is commonly used we have opted to also retain these within the manuscript but appreciate the merits of prediction intervals over I^2^ values, hence their inclusion.

8. **Reviewer**: Please provide more detail on the GRADE synthesis methodology, especially considering GRADE should be used to synthesise the entire body of evidence rather than each individual paper (which is what table 2 suggests)

**Response**: The Grade synthesis used was determine the certainty of results obtained for each patient reported outcome measure utilised in the analysis. Decisions made regarding each domain were reached via discussion and analysis of each study by authors AP and DP. Where there was disagreement or uncertainty, an opinion from senior author RSA was sought. Table 2 demonstrates this process being conducted for each outcome measure individually and include information relating to the number of studies which included this measure as well as the number of patients for whom a result was obtained. Further information on this process added to manuscript as shown by tracked changes.

9. **Reviewer**: Suggest using separate risk of bias tool for RCTs

Response: Thank you for this suggestion, risk of bias for included RCTs has been conducted using ROB2 tool and outlined within methods section.

10. **Reviewer**: Was there any plan for narrative synthesis, or only meta-analysis?

**Response**: From the outset our plan was purely for meta-analysis.

**Reviewer 2:**

1. **Reviewer**: Discuss substantial clinical benefit, which was initially proposed by Glassman et al. for the PROMS as the absence of SCB in the selected studies is a limitation.The CORR study by Lyman et al : What Are the Minimal and Substantial Improvements in the HOOS and KOOS and JR Versions After Total Joint Replacement? Clin Orthop Relat Res. 2018 Dec;476(12):2432-2441 mentions (MCID) using distribution- and anchor-based approaches and the difference that can be considered a large improvement in joint health (substantial clinical benefit) using an anchor-based approach.Surprisingly this article is also missing from the references and the findings of this article are very relevant to this review.

**Response**: We would like to thank you for drawing this analysis method to our attention and agree its use and context is of value to this review and research area as a whole. Therefore a passage discussing substantial clinical benefit, its merits and potential use within the field has been added.

2. **Reviewer**: A funnel plot is highly desirable to check for publication bias.

**Response**: Thank you for this suggestion, funnel plots have been added to the results section of the manuscript.
